# MNI-FTD templates, unbiased average templates of frontotemporal dementia variants

**DOI:** 10.1038/s41597-021-01007-5

**Published:** 2021-08-24

**Authors:** Mahsa Dadar, Ana L. Manera, Vladimir S. Fonov, Simon Ducharme, D. Louis Collins

**Affiliations:** 1 McConnell Brain Imaging Centre, Montreal Neurological Institute, McGill University, Montreal, Quebec (QC) Canada; 2 CERVO Brain Research Center, Centre intégré universitaire santé et services sociaux de la Capitale Nationale, Québec, QC Canada; 3 Douglas Mental Health University Institute, Department of Psychiatry, 6875 Boulevard LaSalle, Montreal, QC H4H 1R3 Canada

**Keywords:** Brain, Biomarkers, Dementia

## Abstract

Standard templates are widely used in human neuroimaging processing pipelines to facilitate group-level analyses and comparisons across subjects/populations. MNI-ICBM152 template is the most commonly used standard template, representing an average of 152 healthy young adult brains. However, in patients with neurodegenerative diseases such as frontotemporal dementia (FTD), high atrophy levels lead to significant differences between individuals’ brain shapes and MNI-ICBM152 template. Such differences might inevitably lead to registration errors or subtle biases in downstream analyses and results. Disease-specific templates are therefore desirable to reflect the anatomical characteristics of the populations of interest and reduce potential registration errors. Here, we present MNI-FTD136, MNI-bvFTD70, MNI-svFTD36, and MNI-pnfaFTD30, four unbiased average templates of 136 FTD patients, 70 behavioural variant (bv), 36 semantic variant (sv), and 30 progressive nonfluent aphasia (pnfa) variant FTD patients and a corresponding age-matched template of 133 controls (MNI-CN133), along with probabilistic tissue maps for each template. Public availability of these templates will facilitate analyses of FTD cohorts and enable comparisons between different studies in an appropriate common standardized space.

## Abstract (summary)

**Table:** 

Measurement(s)	average brain template
Technology Type(s)	T1-weighted magnetic resonance imaging
Factor Type(s)	sex • age • education • Clinical Dementia Rating
Sample Characteristic - Organism	Homo sapiens

Machine-accessible metadata file describing the reported data: 10.6084/m9.figshare.14974848

## Background & Summary

Most brain image processing pipelines use average templates as a target for registration, to enable use of prior anatomical information and to obtain a common coordinate system based on which they can perform group level analyses and comparisons^1–4^. The MNI-ICBM152 is the most commonly used average template in the neuroimaging literature. However, in certain populations such as pediatric cohorts or patients with neurodegenerative diseases, the variations between the individual brains and the standard MNI-ICBM152 template of young adults might hinder registration accuracy and lead to increase in registration errors^5^. In addition, an ill-matched template may give rise to subtle biases in registration that affect processed results. Age appropriate and disease specific templates are therefore desirable not only to reflect the overall anatomical differences between the populations of interest and average young healthy adult brains, but also to reduce potential registration errors and biases when processing data from such populations.

Frontotemporal dementia (FTD) is a clinical categorization describing a heterogenous group of progressive neurodegenerative clinical syndromes associated with atrophy of the frontal and/or anterior temporal lobes. FTD represents about 5% of all cases of dementia in unselected autopsies and, together with Alzheimer’s Disease (AD), it is one of the most common causes of early-onset dementia^6^. FTD is divided into three major clinical syndromes: the behavioral variant (bvFTD) characterised by prominent early behavioral and personality changes, and the two language variants: the semantic variant (svFTD) and the non-fluent primary progressive aphasia (pnfaFTD). The language variants, also known together as primary progressive aphasias, show language deficits (production, naming, syntax or comprehension) as the main symptom at disease onset without remarkable behavioral disturbance^7^. A third language variant, the logopenic PPA characterized by prominent hesitations and word retrieval problems, is often included in the FTD umbrella, but it is pathologically most often associated to AD as opposed to frontotemporal lobar degeneration.

Due to the remarkable heterogeneity in FTD neuropathology, as well as the syndromic overlap with other dementias and psychiatric disorders, a confirmed diagnosis within the FTD spectrum is often difficult to achieve in the absence of a dominant genetic mutation (which represents the majority of cases). Though sharing some similarities, the patterns of atrophy differ between the clinical phenotypes^8^. At the group level, bvFTD typically shows frontal and temporal involvement (particularly, prefrontal cortex, anterior temporal regions, insula, anterior cingulate, striatum and thalamus). However, individual findings may differ on degree of asymmetry, the predominance frontal or temporal involvement, or the extent of posterior cortical atrophy. The two language variants share left side predominance. While, svFTD shows primarily anterior and inferior temporal atrophy, distribution of atrophy in pfnaFTD frequently involves the inferior frontal gyrus, dorsolateral prefrontal cortex, superior temporal gyrus and insula. These differences have in fact been used to classify FTD variants at individual subject level, with accuracies ranging between 72–91%^9–14^. Therefore, brain imaging with magnetic resonance imaging (MRI) is paramount to increase the level of diagnostic confidence. While it is still not part of standard clinical practice, the potential value of morphometric MRI analysis for diagnostic purposes has been extensively demonstrated^12,15^. Hence, improving registration accuracy using disease specific templates could allow better characterization of the pattern of atrophy and its change over time, which could be a valuable resource for both single subject diagnosis and surrogate imaging outcome in clinical trials.

We present MNI-FTD136, MNI-bvFTD70, MNI-svFTD36, and MNI-pnfaFTD30, unbiased average templates of the entire FTD cohort as well as templates of the three variants of FTD, respectively. We also present an average template of age-matched control participants scanned with similar parameters (MNI-CN133) at an isotropic resolution of 1 × 1 × 1 mm^3^. We also include their corresponding probabilistic tissue maps for grey matter (GM), white matter (WM), and cerebrospinal fluid (CSF), generated through automatic segmentation. The public availability of these templates will facilitate analysis of FTD cohorts and enable comparisons between different studies in a common standardized space appropriate to FTD populations.

## Methods

### Data

The frontotemporal lobar degeneration neuroimaging initiative (FTLDNI) was funded through the National Institute of Aging and started in 2010. The primary goals of FTLDNI are to identify neuroimaging modalities and methods of analysis for tracking frontotemporal lobar degeneration (FTLD) and to assess the value of imaging versus other biomarkers in diagnostic roles. FTLDNI is the result of collaborative efforts at three sites in North America (site 1, site 2 and site 3). For up-to-date information on participation and protocol, please visit: http://memory.ucsf.edu/research/studies/nifd. FTLDNI data are publicly available for the purposes of scientific investigation, teaching, or planning of clinical research studies. Researchers can request access through (https://ida.loni.usc.edu/login.jsp or https://ida.loni.usc.edu/collaboration/access/appLicense.jsp;jsessionid=05F0EDD2E8E00292830DC9AB63DA3155).

Data were accessed and downloaded through the LONI platform in August 2018. We included baseline data from FTD (N_bvFTD_ = 70, N_svFTD_ = 36, N_pnfaFTD_ = 30) patients and age-matched control participants (N_Control_ = 133) from the FTLDNI database who had T1-weighted (T1w) MRI scans available. All subjects provided informed consent and the protocol was approved by the institution review board at all sites. Table 1 provides the demographic information for the participants in each group.

**Table 1:** Demographic characteristics of the FTLDNI participants.

Measure	MNI-bvFTD70	MNI-svFTD36	MNI-pnfaFTD30	MNI-CN133
N	70	36	30	133
N_Female_	26	16	15	56
Age	61.71 ± 6.25	62.82 ± 6.26	67.76 ± 7.78	63.94 ± 7.56
Education	15.77 ± 3.31	17.26 ± 3.05	16.03 ± 2.88	17.62 ± 1.86
CDR	1.15 ± 0.63	0.70 ± 0.32	0.56 ± 0.59	0.02 ± 0.11

Data are numbers (N) or mean ± standard deviation. CDR = Clinical Dementia Rating.

### Preprocessing

All baseline T1w scans were pre-processed in three steps: image denoising^16^, intensity non-uniformity correction^17^, and image intensity normalization into a 0–100 range. The pre-processed images were then linearly^18^ registered to the MNI-ICBM152-2009c template^19^. Brain extraction was then performed using the registered images^20^. The quality of the registrations and brain masks were visually assessed to ensure they were accurate.

### Template Generation

**Fig. 1:**
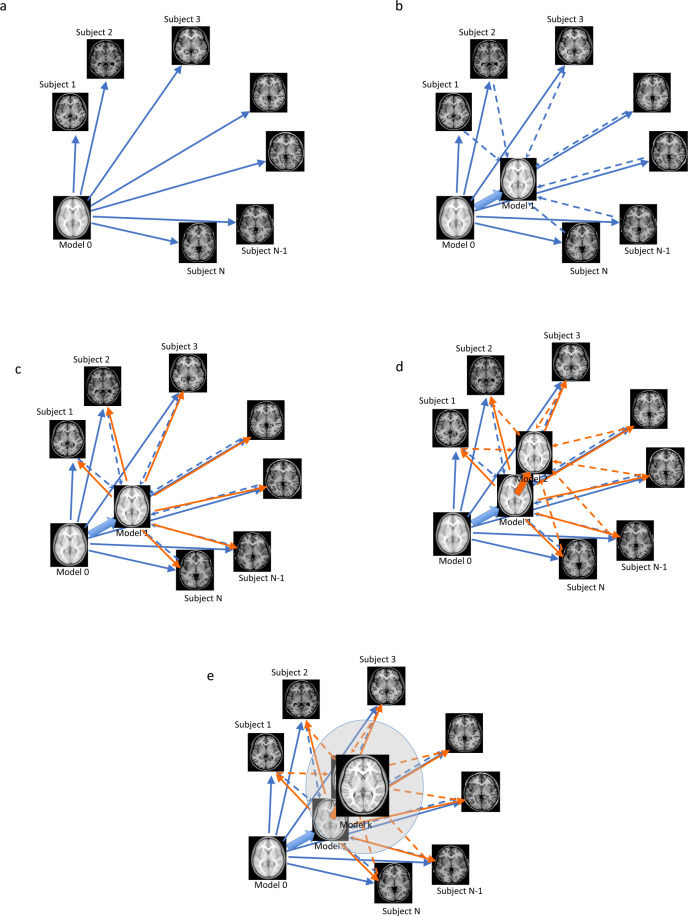
Schematic representation of the unbiased template creation procedure by Fonov *et al*. 2011. (**a**) the initial model (Model 0) is mapped to each subject. (**b**) Mapping of individual subjects to the next model (Model 1). (**c**) the new model is mapped to each subject. (**d**) Mapping of individual subjects to the next model (Model 2). (**e**) The process is iteratively repeated until the final model is generated.

A previously validated method was used to generate unbiased average templates for the entire FTD cohort, as well as the bvFTD, svFTD, pnfaFTD subgroups and the age-matched control participants^5,21^. In short, the method implements a hierarchical nonlinear registration procedure using Automatic Nonlinear Image Matching and Anatomical Labelling (ANIMAL)^22^, reducing the step size at each iteration until convergence is reached. This use of iterative nonlinear registrations in the template generation process leads to average brains that reflect the anatomical characteristics of the population of interest while achieving higher levels of anatomical detail^21^. Figure 1 shows the schematics of the template creation procedure. For all the templates, a symmetric version was also generated to enable assessment of the extent of asymmetry (i.e. differences in the left and right hemispheres) in each template^5,21^. In addition to MNI-CN133, which was generated based on all available control subjects (matched to the entire FTD cohort), we generated three additional control templates, each based on the same number of subjects in each variant (e.g. 70 for bvFTD, 37 for svFTD, and 30 for pnfaFTD), and age and sex matched to the population in that specific variant.

**Fig. 2:**
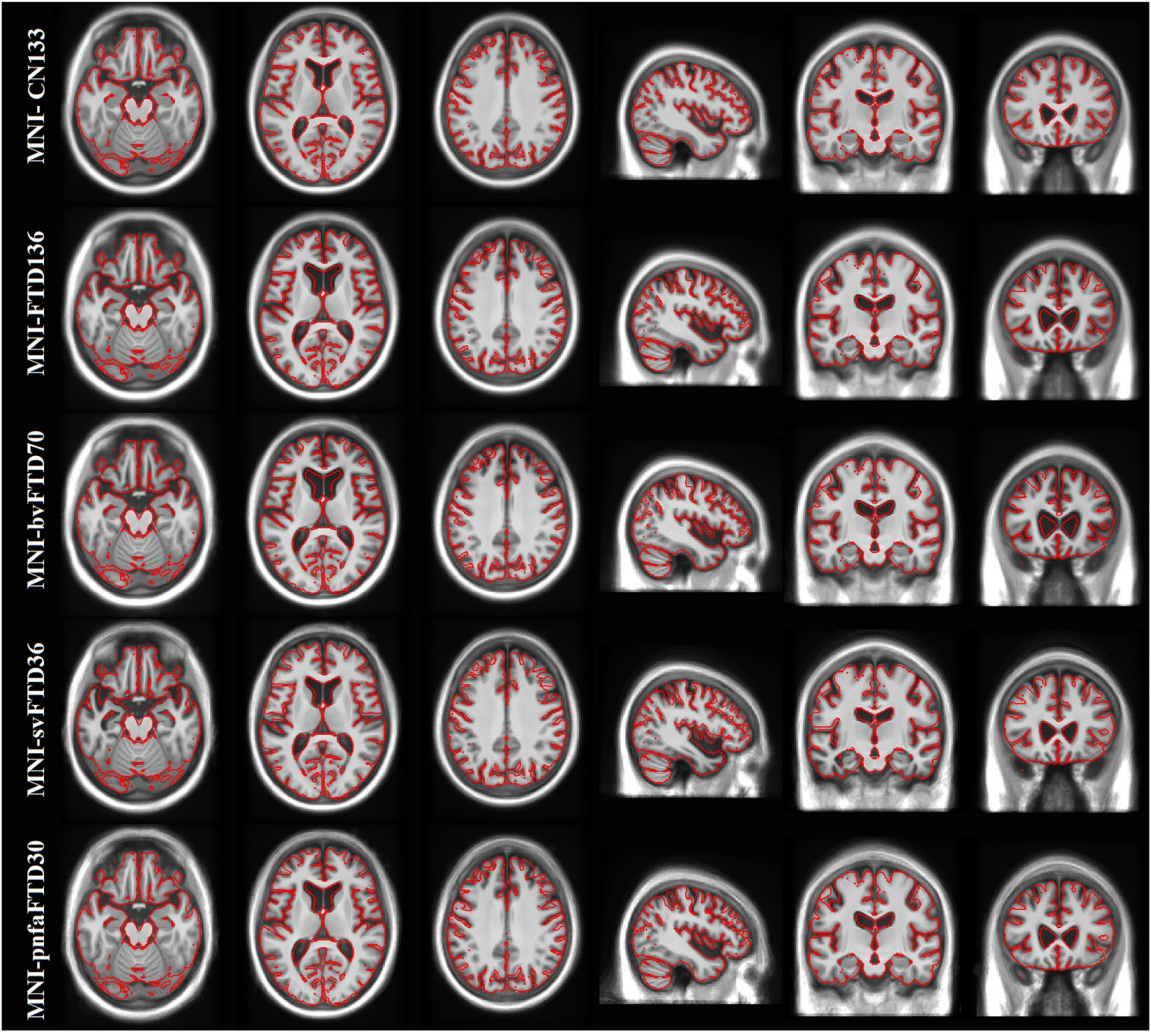
MNI-CN133, MNI-FTD136, MNI-bvFTD70, MNI-svFTD36, and MNI-pnfaFTD30 average templates, overlaid by the contours of the MNI-CN133 template. The figure shows the predominant anterior frontal atrophy compared to controls, which is more evident for the MNI-bvFTD70, whilst MNI-svFTD36 template shows preponderant left anterior temporal atrophy.

Figure 2 presents axial, sagittal, and coronal slices of each template covering the brain, overlaid by the tissue contours of the MNI-CN133 template to highlight their differences (also see Figure S1 in the supplementary materials, in which arrows point to the main areas of atrophy in each FTD template). The FTD template shows bilateral but asymmetric fronto-insular, anterior temporal and lateral temporal atrophy compared to the age-matched healthy controls (MNI-CN133 template). The bvFTD template shows a similar pattern, but with more evident atrophy on subcortical structures together with greater ventricle enlargement, mainly in the frontal horns of the lateral ventricles. A predominantly left sided temporal atrophy pattern with corresponding ventricular enlargement of the temporal horns is shown on the svFTD template. Finally, the pnfaFTD presents with preponderantly left frontal atrophy with evident enlargement of the frontal horns of the lateral ventricles, though less significant than bvFTD. Figures S2–S5 in the supplementary materials show the symmetric and asymmetric average templates for each FTD variant, along with their corresponding age and sex matched control template.

### Tissue Maps

**Fig. 3:**
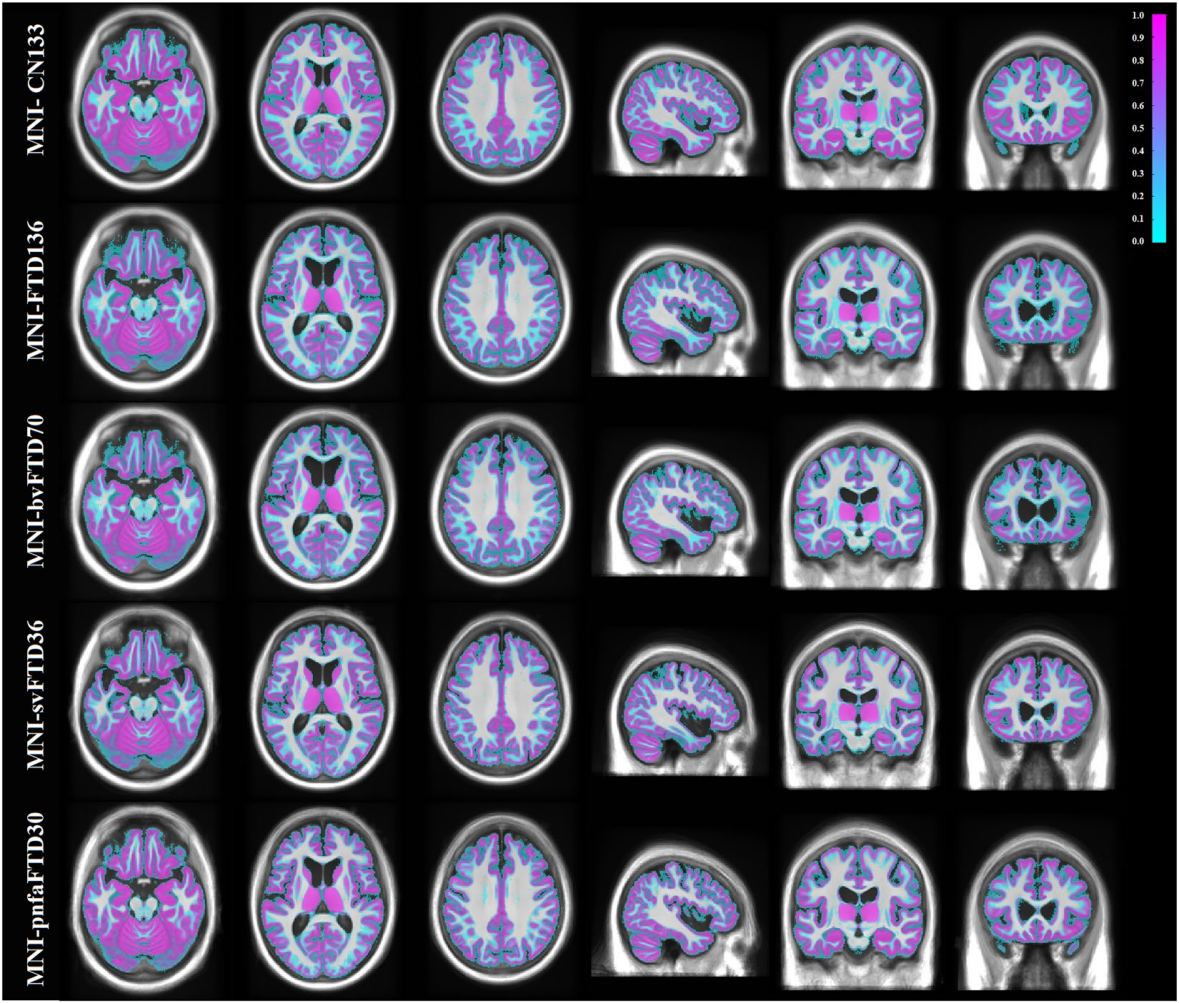
Grey matter probability maps overlaid on the average templates.

**Fig. 4:**
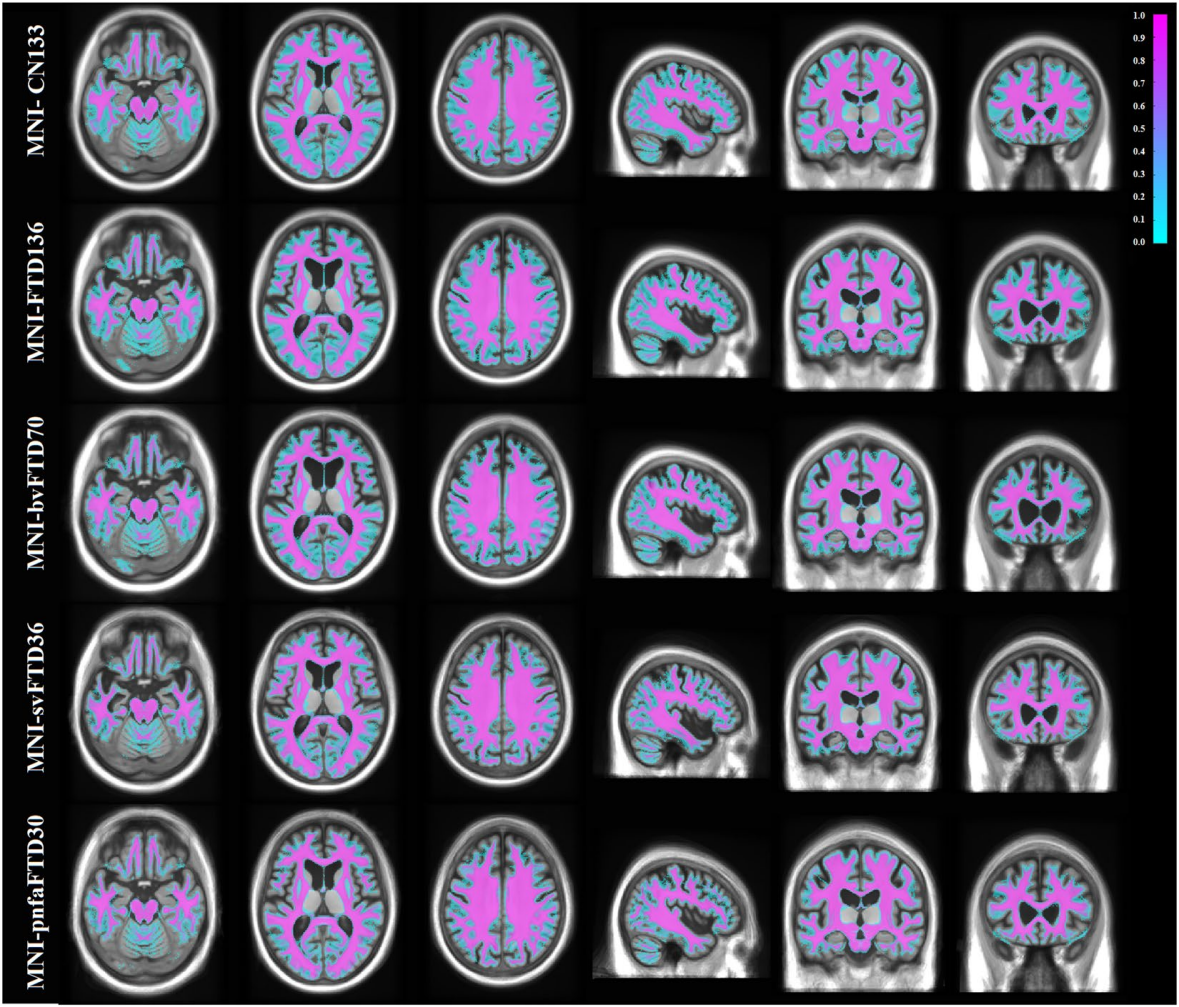
White matter probability maps overlaid on the average templates.

**Fig. 5:**
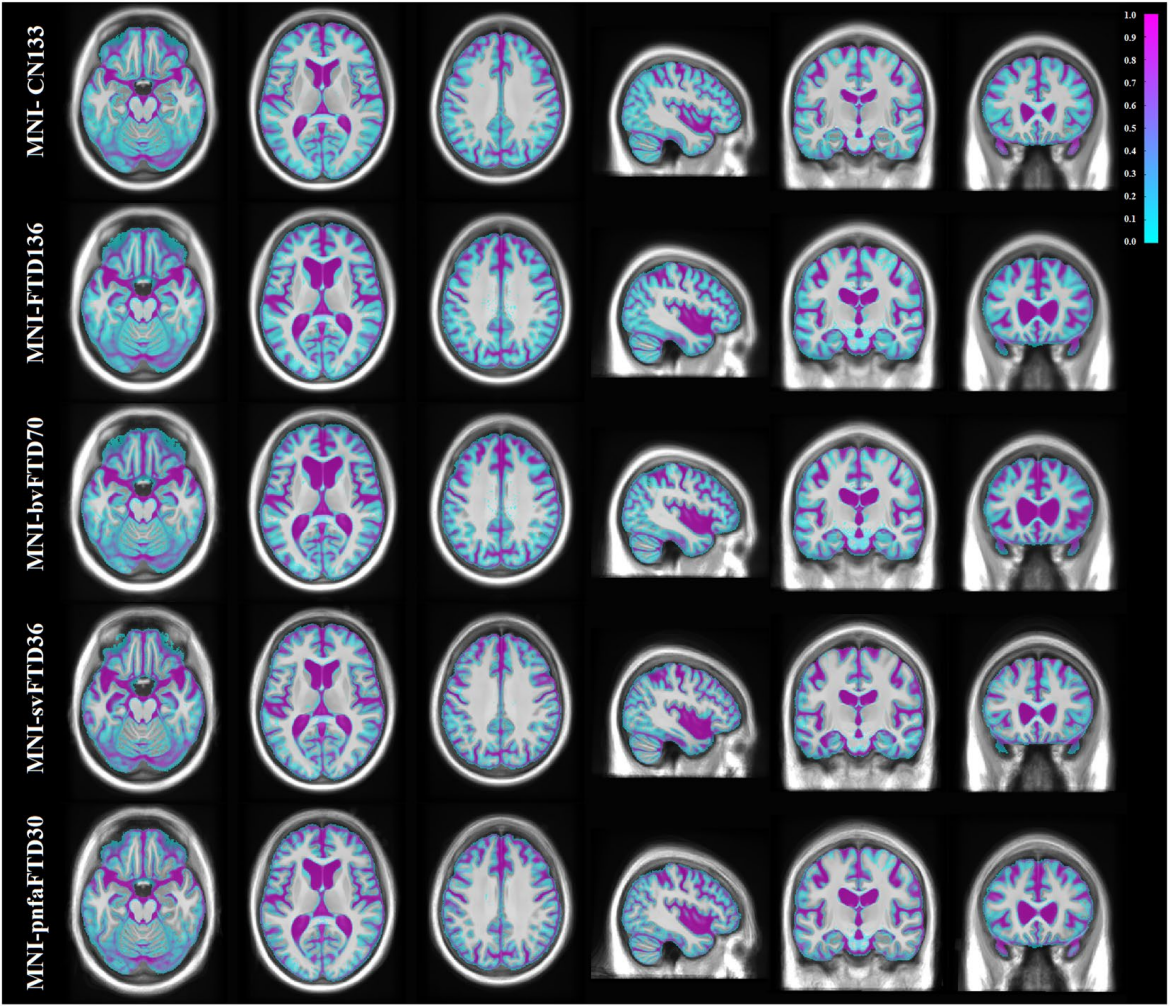
CSF probability maps overlaid on the average templates.

Cortical GM, WM, and CSF were automatically segmented for each individual subject using the BISON tissue classification tool, developed and extensively validated for use in multi-center and multi-scanner datasets of aging and neurodegenerative diseases^23,24^. Deep GM structures (i.e. putamen, caudate, thalamus, and pallidum) were also segmented using a previously validated deep convolutional neural network (CNN) method^25^. The method has been developed and validated using Neuromorphometrics dataset (http://www.neuromorphometrics.com), which includes subjects aged between 5 to 96 years, with varying levels of atrophy. The method showed excellent agreement against manual segmentations (mean Dice similarity index = 0.9), providing robust and reliable results in the multi-center and multi-scanner Neuromorphometrics dataset. The resulting GM, WM, and CSF segmentations from each subject were then nonlinearly resampled to their appropriate templates using the final subject-to-template transform computed in the creation of the unbiased template (e.g. the tissue labels from bvFTD patients were aligned to the MNI-bvFTD70 template). Probabilistic tissue maps were then generated by averaging the nonlinearly registered tissue labels for each cohort. The quality of the segmentations was visually assessed to ensure that only correctly segmented cases were used to create the probabilistic tissue maps. Figures 3–5 show the tissue maps overlaid on their corresponding templates for GM, WM, and CSF.

### Transformations between Template Pairs

To enable transformations between each template pair and also between each template and the commonly used MNI-ICBM-2009c template, we nonlinearly registered each template to all other templates as well as MNI-ICBM-2009c template, using advanced normalization tools (ANTs) diffeomorphic registration tool.

## Data Records

The symmetric and asymmetric average templates (i.e. MNI-FTD136, MNI-bvFTD70, MNI-svFTD36, MNI-pnfaFTD30, and MNI-CN133) as well as their corresponding tissue maps, and nonlinear transformations between each template pair as well as transformations from each template to MNI-ICBM152-2009c are available at G-Node (https://gin.g-node.org/anamanera/MNI-FTD_Templates.git)^26^ and http://nist.mni.mcgill.ca/?p=904. All data are available in compressed MINC^27,28^ and NIfTI formats.

## Technical Validation

Prior to generating the average templates, the quality of the images (e.g. presence of image artifacts such as motion) as well as the linear and nonlinear registrations was visually assessed and 16 cases (2 CN, 7 bv FTD, 3 svFTD, and 4 pnfaFTD) that did not pass this quality control step were discarded. Similarly, 10 cases (4 CN, 4 bv FTD, 1 svFTD, and 1 pnfaFTD) failed quality control for tissue segmentation step and were discarded before generating the tissue probability maps. Figures S6–S8 in the supplementary materials show examples of QC images for cases failed due to presence of motion artifact (Figure S6), nonlinear registration failure (Figure S7), and tissue segmentation failure (Figure S8). For comparison, Figures S9, S10, and S11 show examples of passed cases for linear and nonlinear registrations and tissue classification, respectively. No cases failed quality control for linear registration. For more details on the registration quality control procedure, see Dadar *et al*.^18^.

**Fig. 6:**
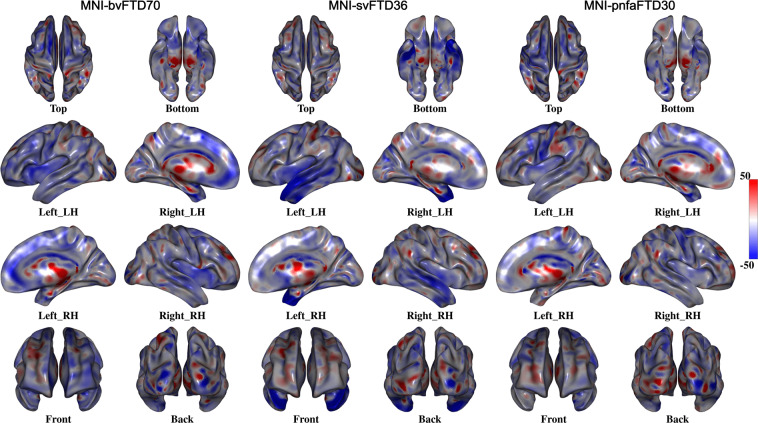
Percentage of cortical thickness difference between MNI-bvFTD70, MNI-svFTD36, and MNI-pnfaFTD30 versus MNI-CN133 templates. MNI-bvFTD70 template shows the largest amount of frontal lobe cortical atrophy. MNI-svFTD36 shows the greatest amount of bi-lateral temporal lobe cortical atrophy compared to the two other FTD variants. MNI-pnfaFTD30 shows left sided cortical thinning in prefrontal and Broca’s areas.

**Fig. 7:**
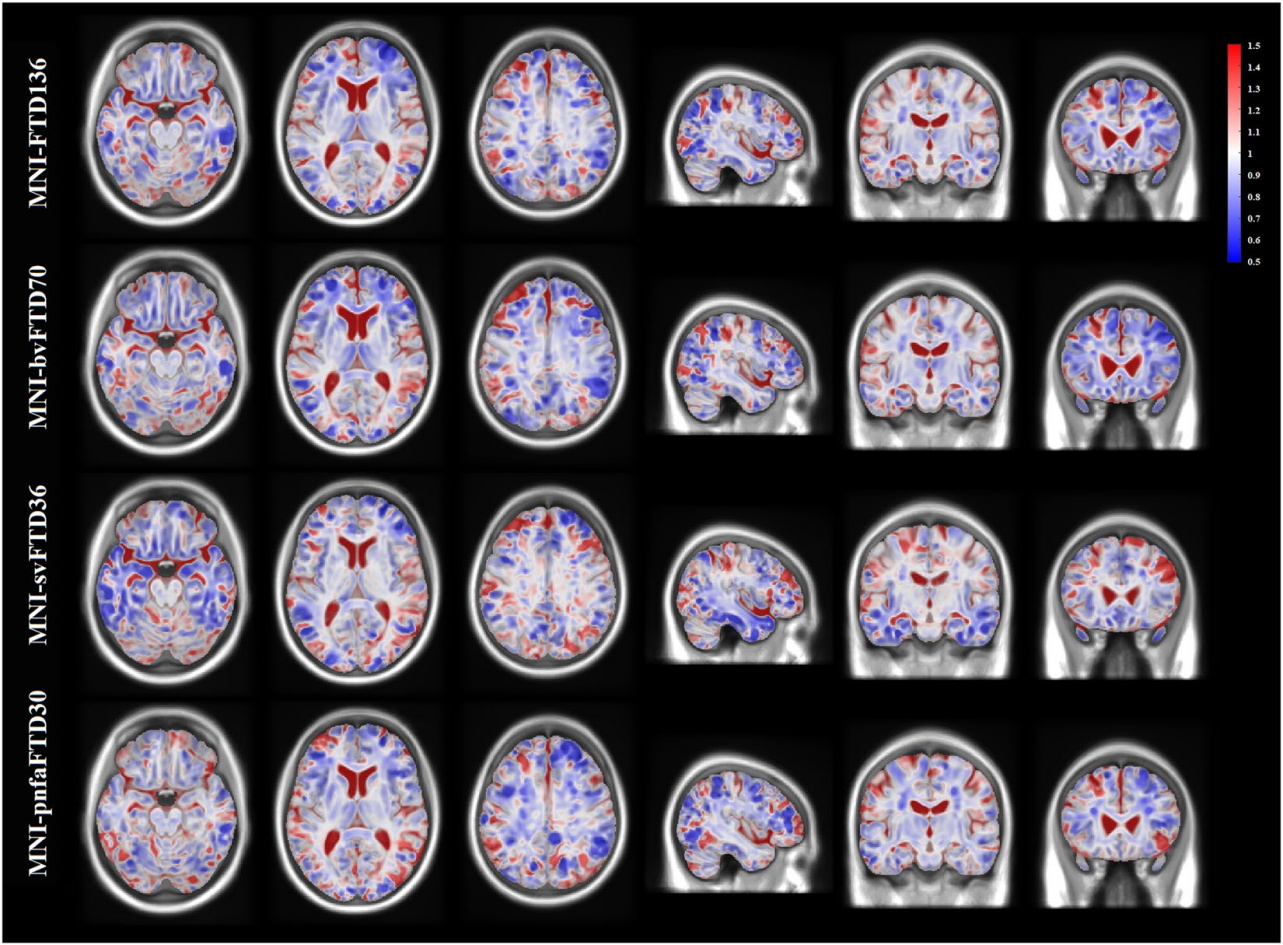
Deformation based morphometry difference between MNI-bvFTD70, MNI-svFTD36, and MNI-pnfaFTD30 versus MNI-CN133 templates. All FTD templates show increased ventricular and sulcal spaces. MNI-bvFTD70 shows predominant bilateral anterior frontal shrinkage. MNI-svFTD36 shows asymmetric in lateral and anterior temporal lobe atrophy. MNI-pnfaFTD30 shows asymmetric frontal lobe atrophy.

To further demonstrate the structural differences between the templates, the FALCON cortical surface extraction tool was applied to each template, and the relative cortical thickness difference between each of the FTD templates and MNI-CN133 was calculated^29^. Similarly, to demonstrate the differences between the templates in the deep gray matter and white matter areas, deformation based morphometry maps were generated based on nonlinear registrations between each FTD template and the MNI-CN133 template^30^. Figure 6 shows the percentage of difference in cortical thickness between MNI-bvFTD70, MNI-svFTD36, and MNI-pnfaFTD30 versus MNI-CN133 cortical surfaces. Colder colors indicate thinner cortex in comparison with MNI-CN133 template. Similarly, Fig. 7 shows the regional differences between MNI-bvFTD70, MNI-svFTD36, and MNI-pnfaFTD30 versus MNI-CN133 across the entire brain. Colder colors indicate smaller areas (i.e. shrinkage or more atrophy), and warmer colors (e.g. in the ventricular and sulci regions) indicate larger areas (i.e. expansion) in comparison with MNI-CN133 template.

Comparing the MNI-bvFTD70 group against the controls, the greatest difference in cortical thickness was located in frontal lobes, bilaterally as well as the temporal poles (specially in medial frontal and dorsolateral prefrontal areas). In MNI-svFTD36, however, cortical thinning is limited to the anterior and lateral temporal lobe, predominantly on the left side. Finally, MNI-pnfaFTD30 shows left sided cortical thinning in prefrontal and Broca’s areas.

Correspondingly, deformation-based morphometry maps show widespread ventricular and sulcal enlargement accompanied by an overall pattern of gray matter atrophy in all FTD templates. In the MNI-FTD136 template, the atrophy was more evident in right anterior frontal and lateral temporal regions. While MNI-bvFTD70 demonstrated a predominant bilateral anterior frontal shrinkage, MNI-pnfaFTD30 showed asymmetric frontal atrophy. As expected, in MNI-svFTD36, smaller areas were located asymmetrically in lateral and anterior temporal lobes. WM atrophy followed the pattern of GM atrophy for all the templates; i.e. evident patterns of atrophy in the frontal and lateral temporal regions in MNI-FTD136, predominant anterior frontal atrophy in MNI-bvFTD70, asymmetric frontal atrophy in MNI-pnfaFTD30, and asymmetric lateral and anterior temporal atrophy in MNI-pnfaFTD30. In addition, there was widespread grey and white matter atrophy in the cerebellar regions in all FTD templates.

The disorders under FTD spectrum (i.e. bv, sv, and pnfa) not only share underlying pathology and heritability, but also overlap significantly in terms of clinical symptoms. It is therefore also expected that they would share some anatomical similarities in atrophy patterns. The latter would explain the very subtle differences amongst the FTD variant templates^31^.

We acknowledge the influence of sex on atrophy burden on many neurodegenerative disorders^32^, including FTD^33–35^. Prior studies on FTD have shown that women present with greater levels of frontotemporal atrophy and cortical thinning at the time of bvFTD diagnosis than men, after accounting for confounding factors. Yet, for a given amount of atrophy, women performed better expected in executive tasks than men but had more apathy, sleep, and behavioral disturbances^33^. Nevertheless, clinical features at diagnosis, disease progression, and survival were similar in men and women. This evidence highlights the relevance of considering sex in the generation of disease specific templates. However, our current sample size was insufficient to generate sex-stratified templates. In addition, education has been shown to facilitate brain reserve and maintenance; i.e. individuals with higher levels of education tend to have better cognition, and slower grey matter volume changes^36,37^. Unfortunately, in the data used in this study, the bvFTD patients were significantly less educated than the control participants as well as the svFTD patients (the differences were marginal for the pnfaFTD group: p = 0.08). These differences might translate into greater atrophy in the bvFTD templates than would have been observed in an education matched cohort. We acknowledge that this might be a limitation of our study, and future studies with education matched cohorts are needed to establish such differences.

To assess the impact of using population appropriate templates on registration accuracy, we nonlinearly registered (using ANTs diffeomorphic registration tool) all the data (i.e. CN, bvFTD, svFTD, and pnfaFTD subjects) once to MNI-ICBM2009c template (based on healthy young adults) and once to their own appropriate templates, and performed visual quality control on the resulting registrations. Table 2 shows the percentage of failed cases for each cohort and template combination. As expected, in all groups, registration failure rates were much lower when registered to their respective population appropriate templates. For examples of failed and passed nonlinear registrations, see Figures S7 and S10 in the supplementary materials.

**Table 2:** Nonlinear registration error rates (% percentage) for registration of subjects to MNI-ICBM2009c template and population appropriate templates.

Template	MNI-CN133	MNI-FTD136	MNI-bvFTD70	MNI-svFTD36	MNI-pnfaFTD30
MNI-ICBM2009c	3.01%	5.15%	5.71%	5.56%	10.00%
Appropriate Template	0.75%	0.00%	0.00%	0.00%	0.00%

To demonstrate that the differences in the templates were not due to differences in the number of subjects used to generate each template, we randomly sampled thirty cases (same as the number of cases in the smallest group, i.e. pnfaFTD) from each of the bvFTD, svFTD, and control groups, and generated three additional templates (MNI-bvFTD30, MNI-svFTD30, and MNI-CN30). Figure S12 in the supplementary materials shows MNI-bvFTD30, MNI-svFTD30, and MNI-CN30 templates along with their counterparts that were generated based on all available data. The FTD30 templates show similar anatomical features, with predominant anterior frontal atrophy compared to controls, more evident for MNI-bvFTD30, while MNI-svFTD30 shows preponderant left anterior temporal atrophy. We also performed the same registration accuracy assessment using these templates as reference, and obtained similar error rates for all groups (i.e. 0.75% for MNI-CN30, and 0.00% for others).

## Supplementary information

Supplementary Materials

## Data Availability

The scripts for generating unbiased average templates are publicly available at https://github.com/vfonov/build_average_model, also re-implemented in Python and publicly available at (see https://github.com/vfonov/nist_mni_pipelines: iplScoopGenerateModel.py as well as examples/synthetic_tests/test_model_creation/scoop_test_nl_sym.py). The scripts for tissue classification tools and FALCON are publicly available at http://nist.mni.mcgill.ca/?p=2148, https://github.com/philnovv/CNN_NeuroSeg/, and https://github.com/NIST-MNI/falcon, respectively. To replicate our results or generate a new average template, the user needs to provide preprocessed T1w images to the pipeline. Raw T1w images can be preprocessed using our standard pipeline available at https://github.com/vfonov/bic-pipelines. Afterwards, either build_average_model.rb or scoop_test_nl_sym.py can be used to generate the average template.
