# Supplementary material for: MNI-FTD templates, unbiased average templates of frontotemporal dementia variants
Source: Sci Data. 2021 Aug 24;8:222. doi: 10.1038/s41597-021-01007-5 (PMC8385071; doi:10.1038/s41597-021-01007-5)
Supplement: Supplementary file 1 — Supplementary Materials [file 41597_2021_1007_MOESM1_ESM.pdf]

Table of Contents

Figure S1. Average templates …………………………………………………………………………………………………… 2

Figure S2. Symmetric and asymmetric MNI-FTD136 templates ……………………………………………………………….. 3

Figure S3. Symmetric and asymmetric MNI-bvFTD70 templates ………………………………………………………………. 4

Figure S4. Symmetric and asymmetric MNI-svFTD36 templates ………………………………………………………………. 5

Figure S5. Symmetric and asymmetric MNI-pnfaFTD30 templates ……………………………………………………………. 6

Figure S.6. Example of failed quality control image (motion artifact) ……………………………………………...…………… 7

Figure S7. Example of failed quality control image (nonlinear registration) …………………………………………………… 8

Figure S8. Example of failed quality control image (tissue classification) ……………………………………………………… 9

Figure S9. Example of passed quality control image (linear registration) ………………………………………………………. 10

Figure S10. Example of passed quality control image (nonlinear registration) ………………………………………………… 11

Figure S11. Example of passed quality control image (tissue classification) ………………………………………………..… 12

Figure S.12. MNI-CN30, MNI-bvFTD30, and MNI-svFTD30 average templates……………………………………………….. 13

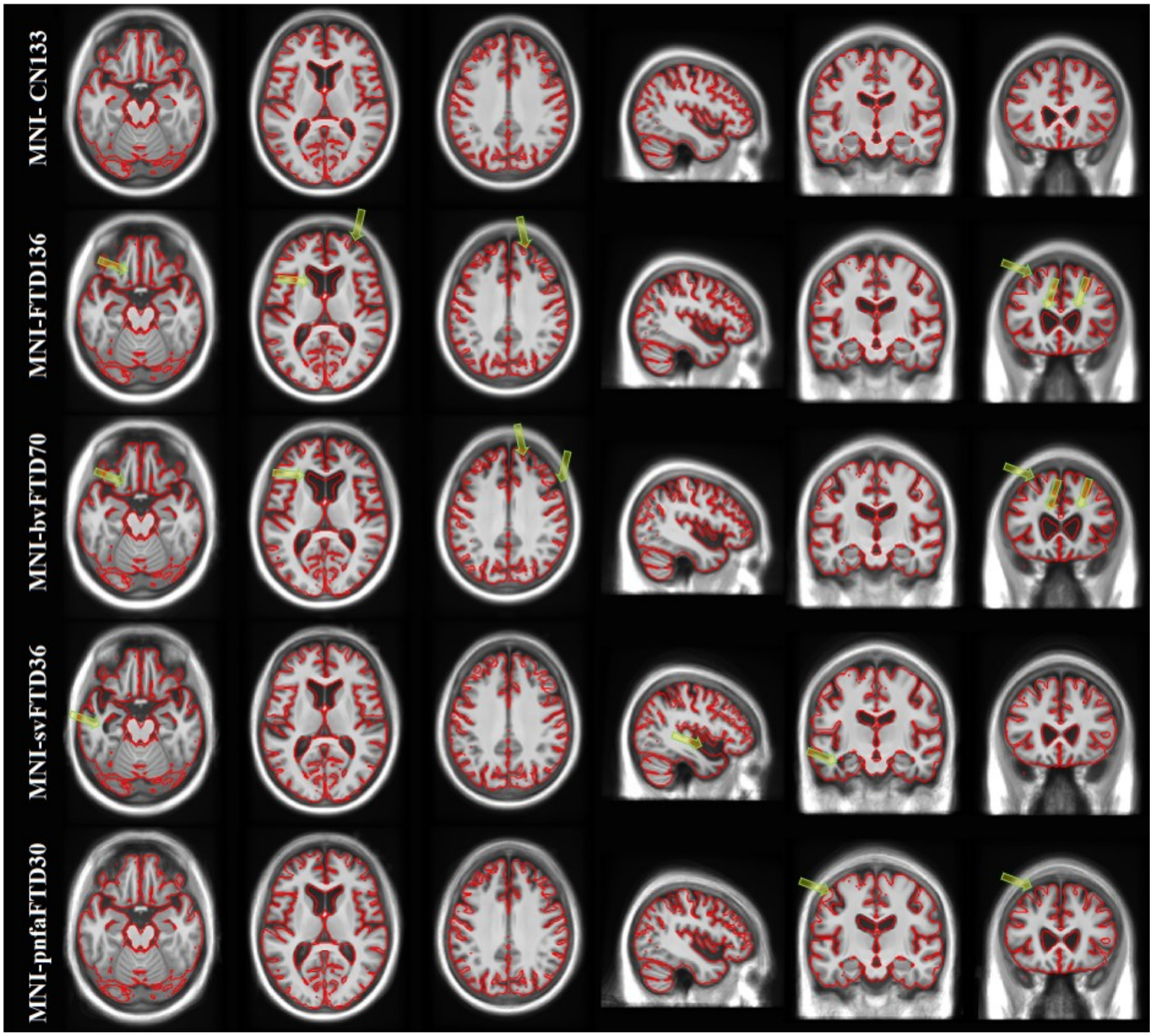

Figure S1. MNI-CN133, MNI-FTD136, MNI-bvFTD70, MNI-svFTD36, and MNI-pnfaFTD30 average templates, overlaid by the contours of the MNI-CN133 template. The figure shows the predominant anterior frontal atrophy compared to controls, which is more evident for the MNI-bvFTD70, whilst MNI-svFTD36 template shows preponderant left anterior temporal atrophy. Arrows point to the areas of atrophy in FTD templates, in comparison to the CN template.

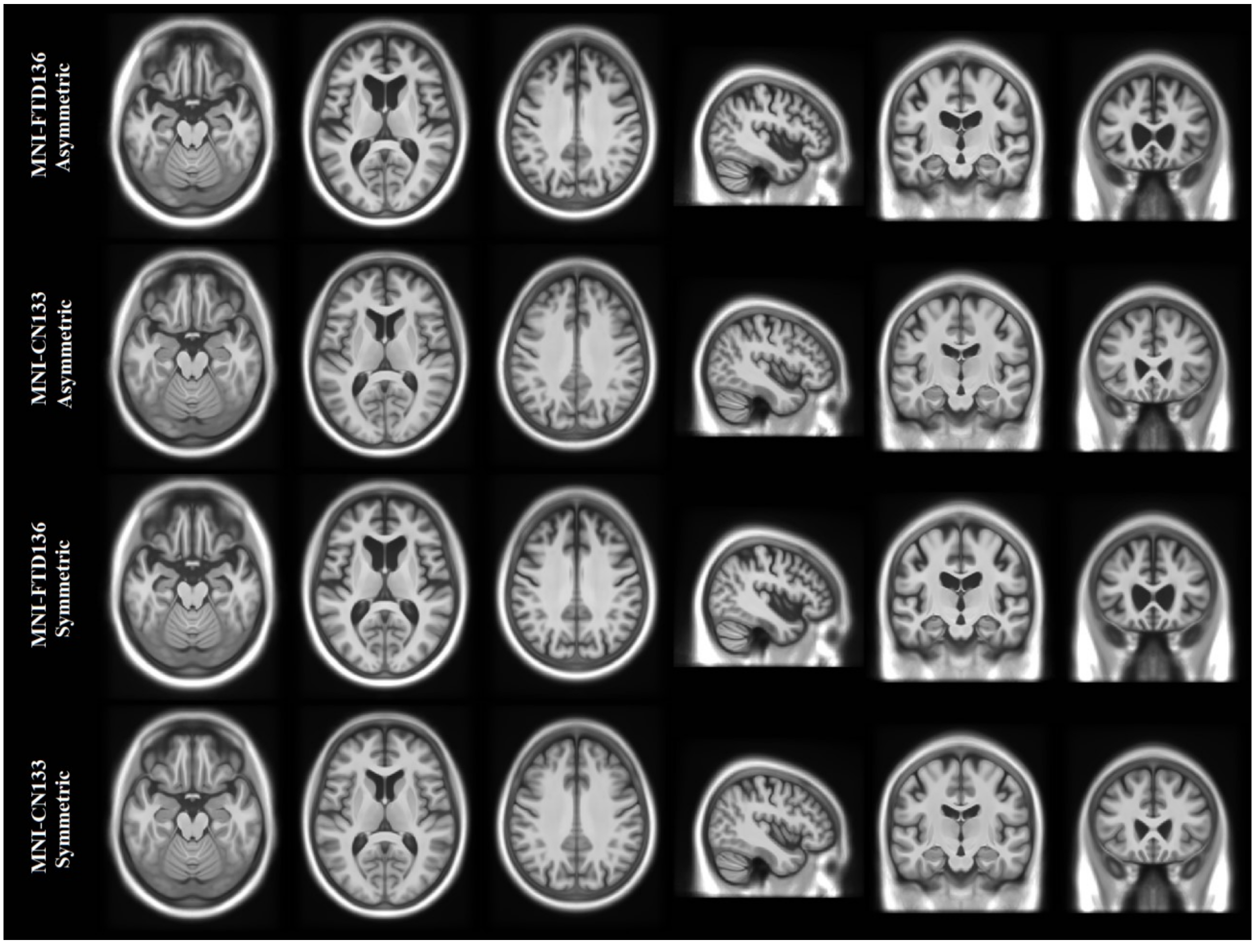

Figure S2. Symmetric and asymmetric MNI-FTD136 templates along with age and sex-matched control templates.

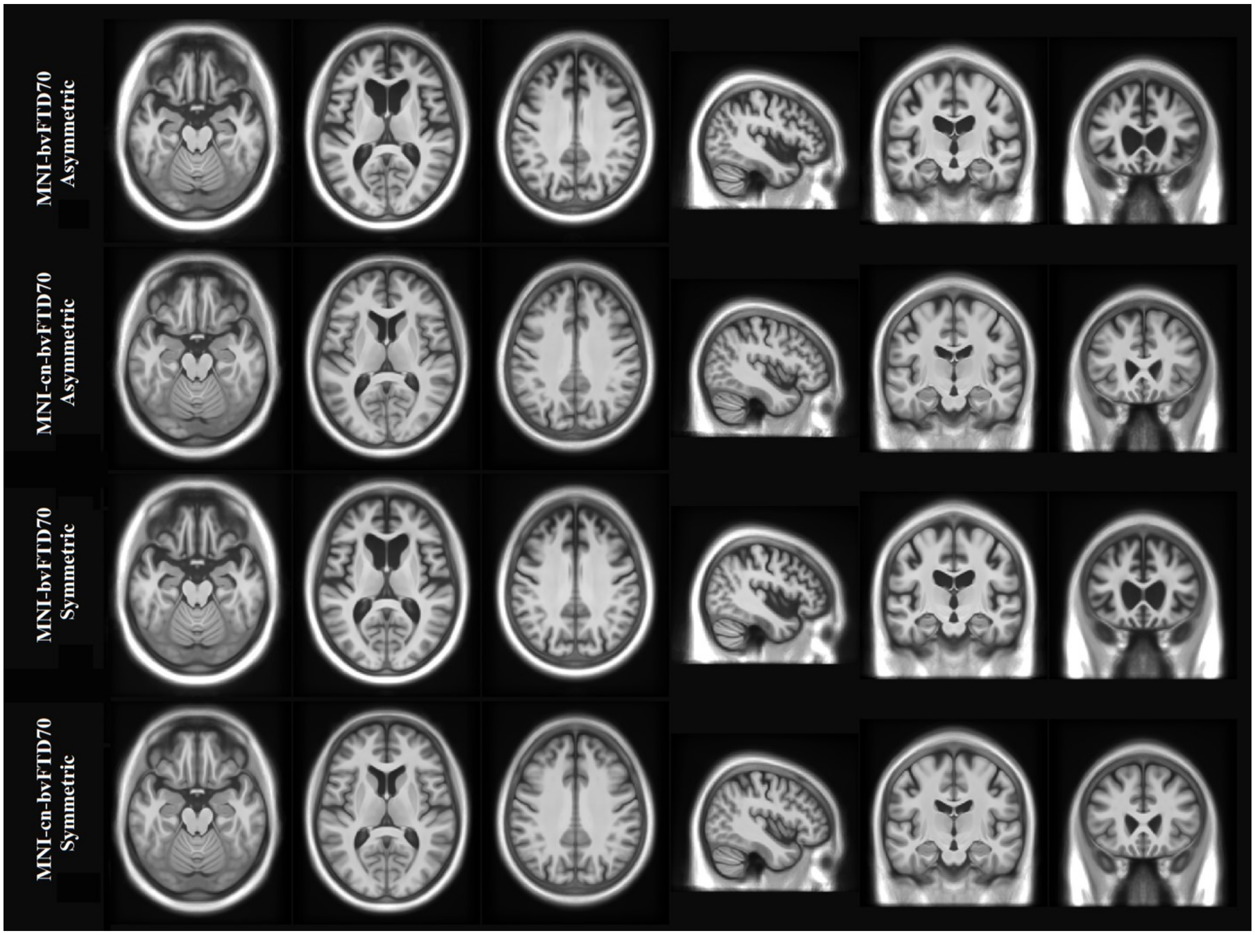

Figure S3. Symmetric and asymmetric MNI-bvFTD70 templates along with age and sex-matched control templates.

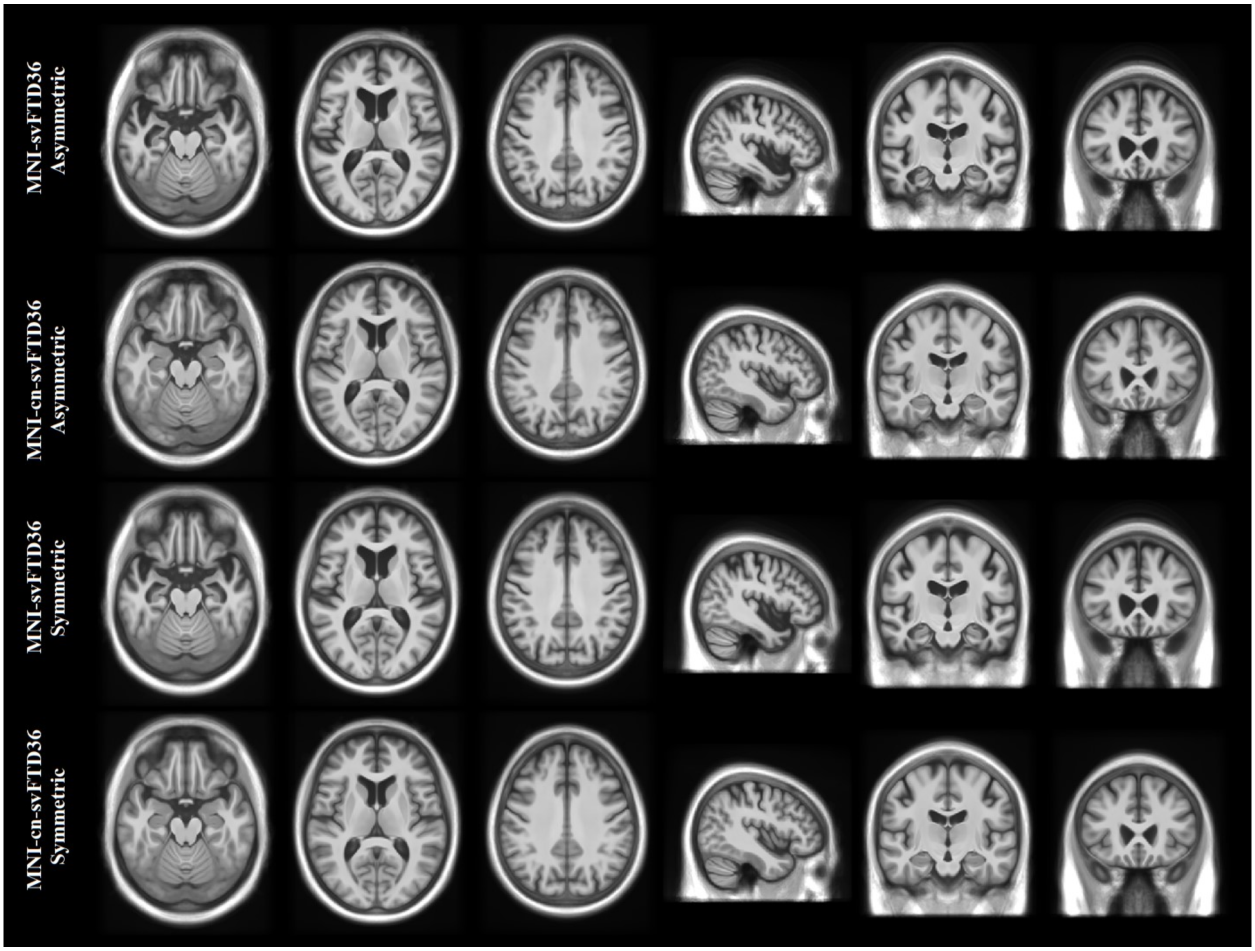

Figure S4. Symmetric and asymmetric MNI-svFTD36 templates along with age and sex-matched control templates.

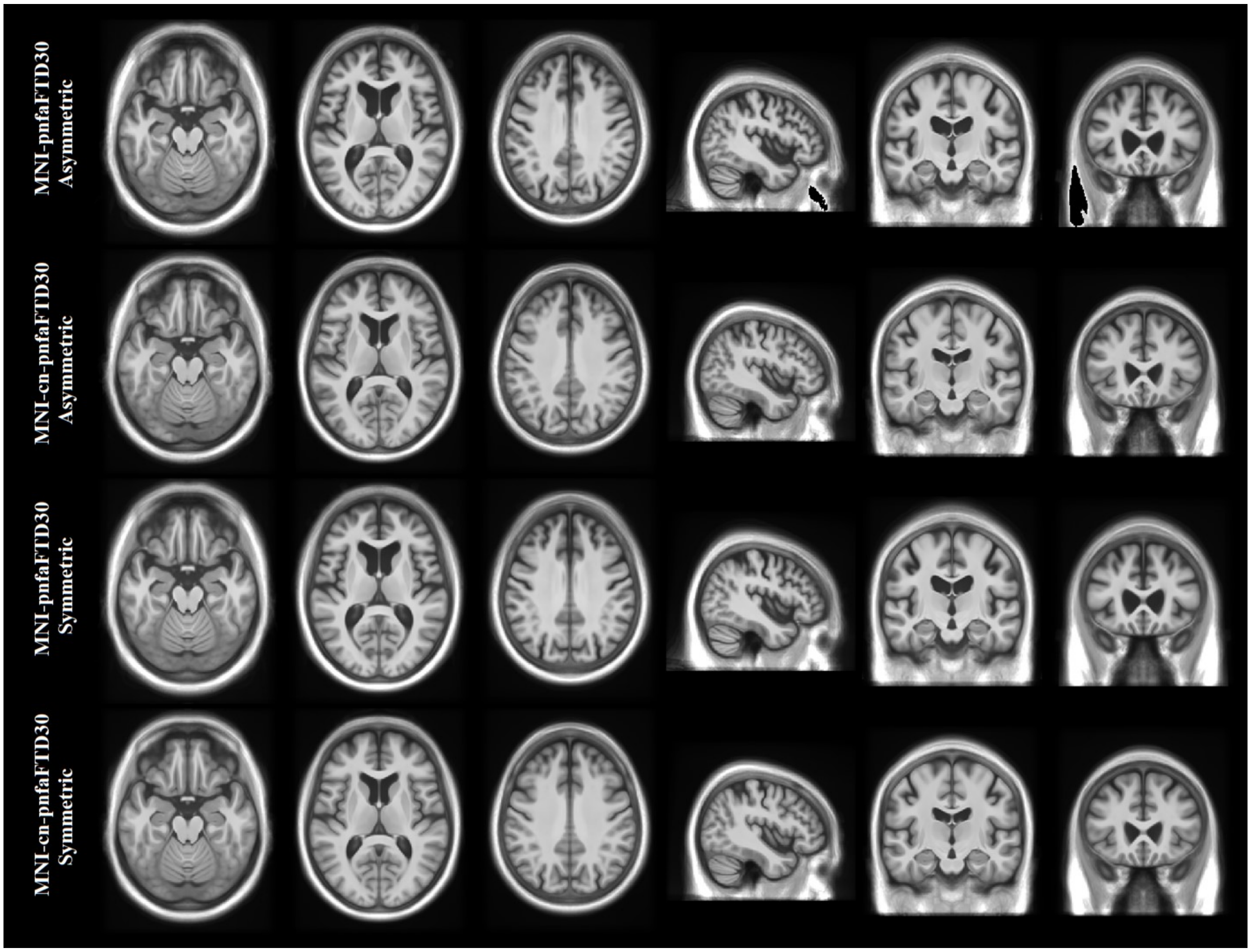

Figure S5. Symmetric and asymmetric MNI-pnfaFTD30 templates along with age and sex-matched control templates.

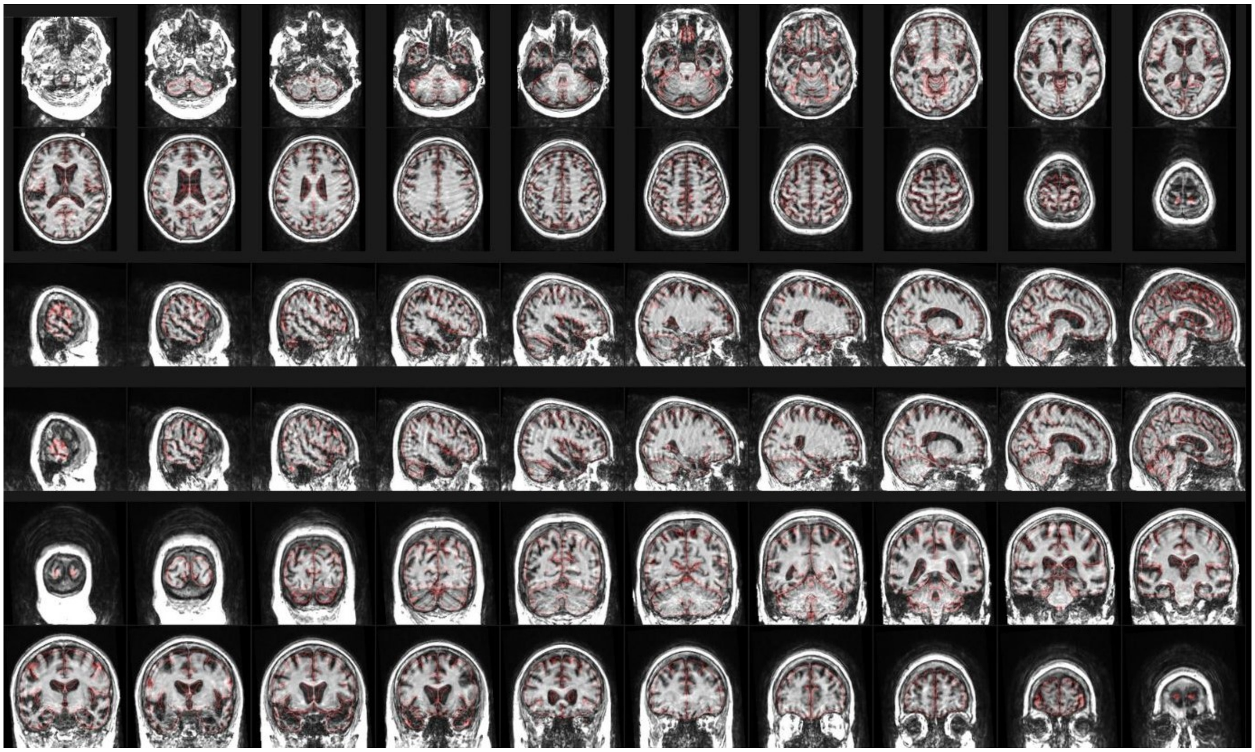

Figure S6. Example of quality control image for a case failed due to presence of motion artifacts.

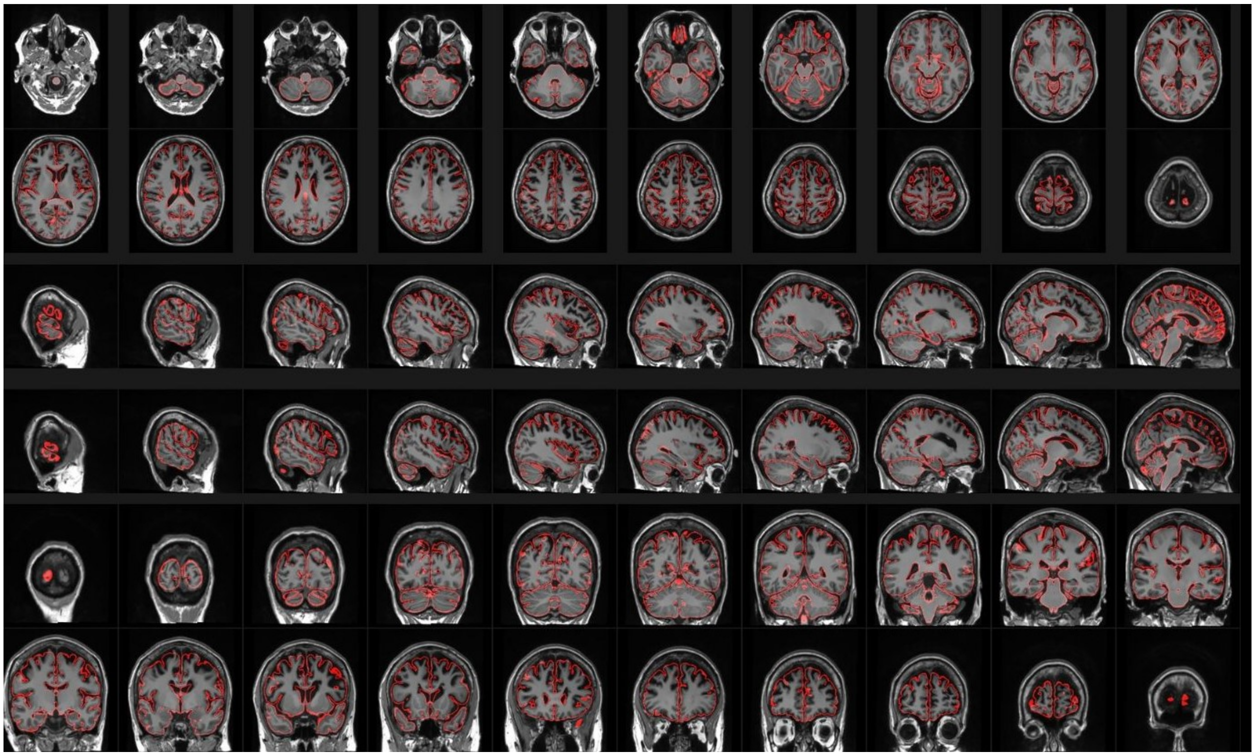

Figure S7. Example of quality control image for a case failed due to inaccurate nonlinear registration. The figure shows the transformed T1w image (after applying the nonlinear transformation) along with the average template contours. The nonlinear registration has failed in the periventricular regions in the frontal lobe.

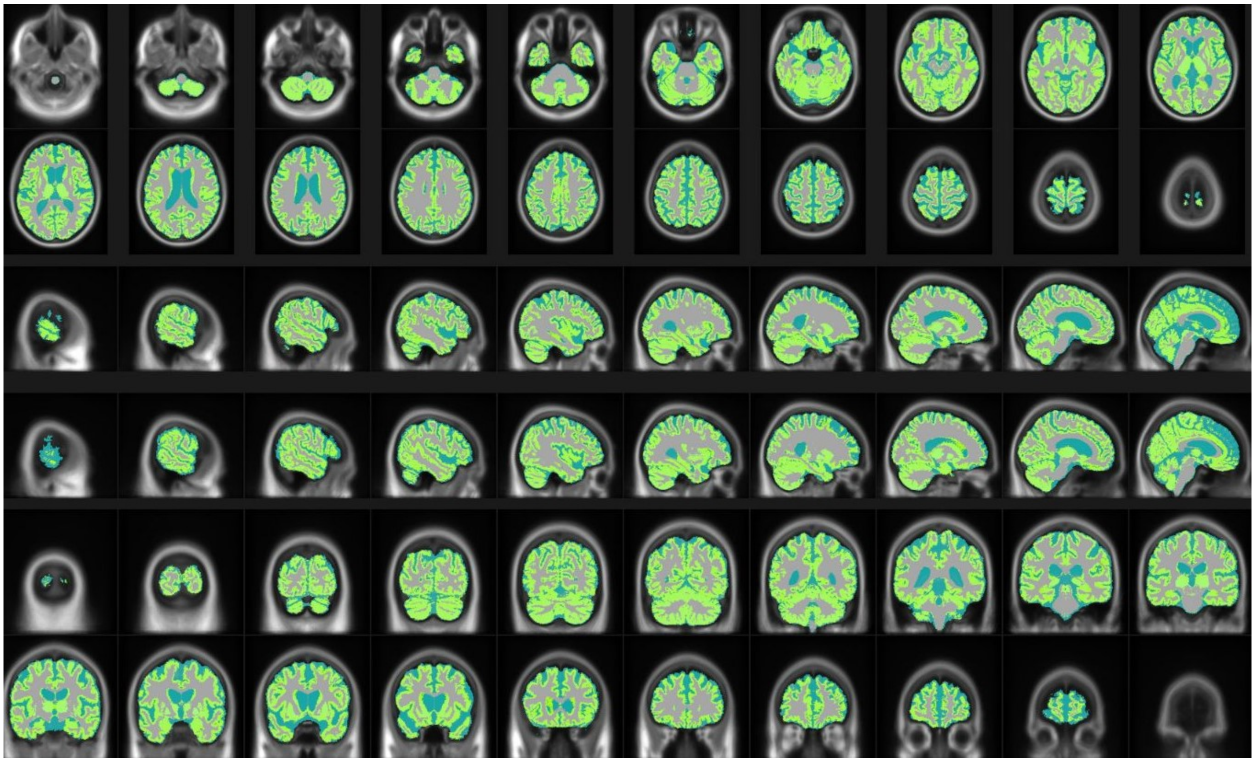

Figure S8. Example of quality control image for a case failed due to inaccurate tissue classification results. Note the area inside the frontal horn of the left ventricle incorrectly segmented as grey matter.

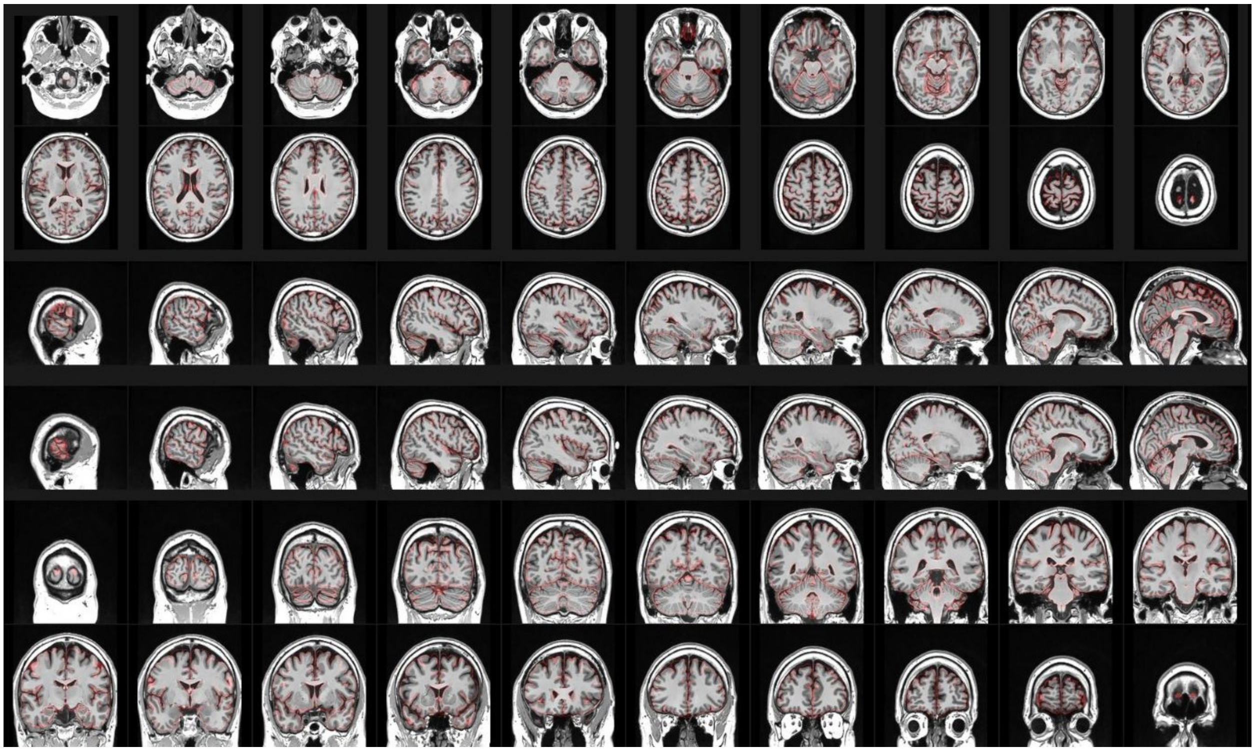

Figure S9. Example of quality control image for a case that passed linear registration quality control.

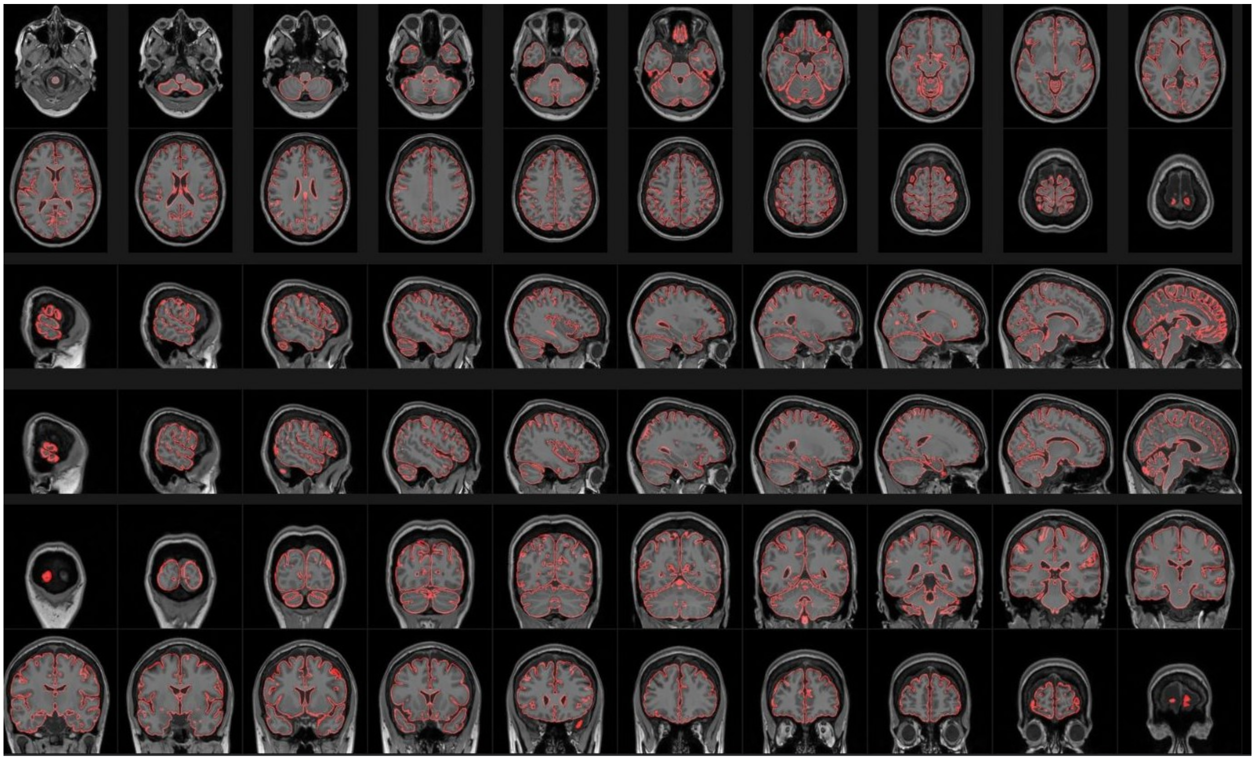

Figure S10. Example of quality control image for a case that passed nonlinear registration quality control. The figure shows the transformed T1w image (after applying the nonlinear transformation) along with the average template contours. Note how the contours of the template match the tissue borders, reflecting i.e. a successful nonlinear mapping.

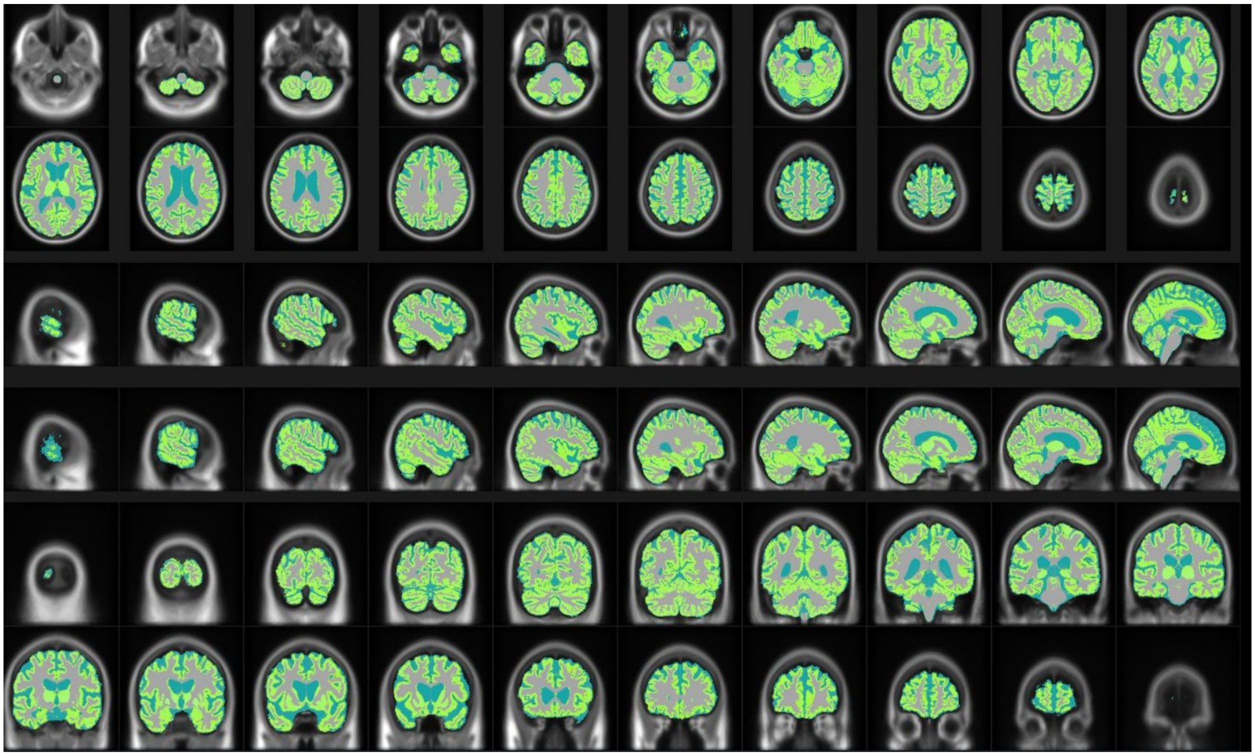

Figure S11. Example of quality control image for a case that passed tissue segmentation quality control.

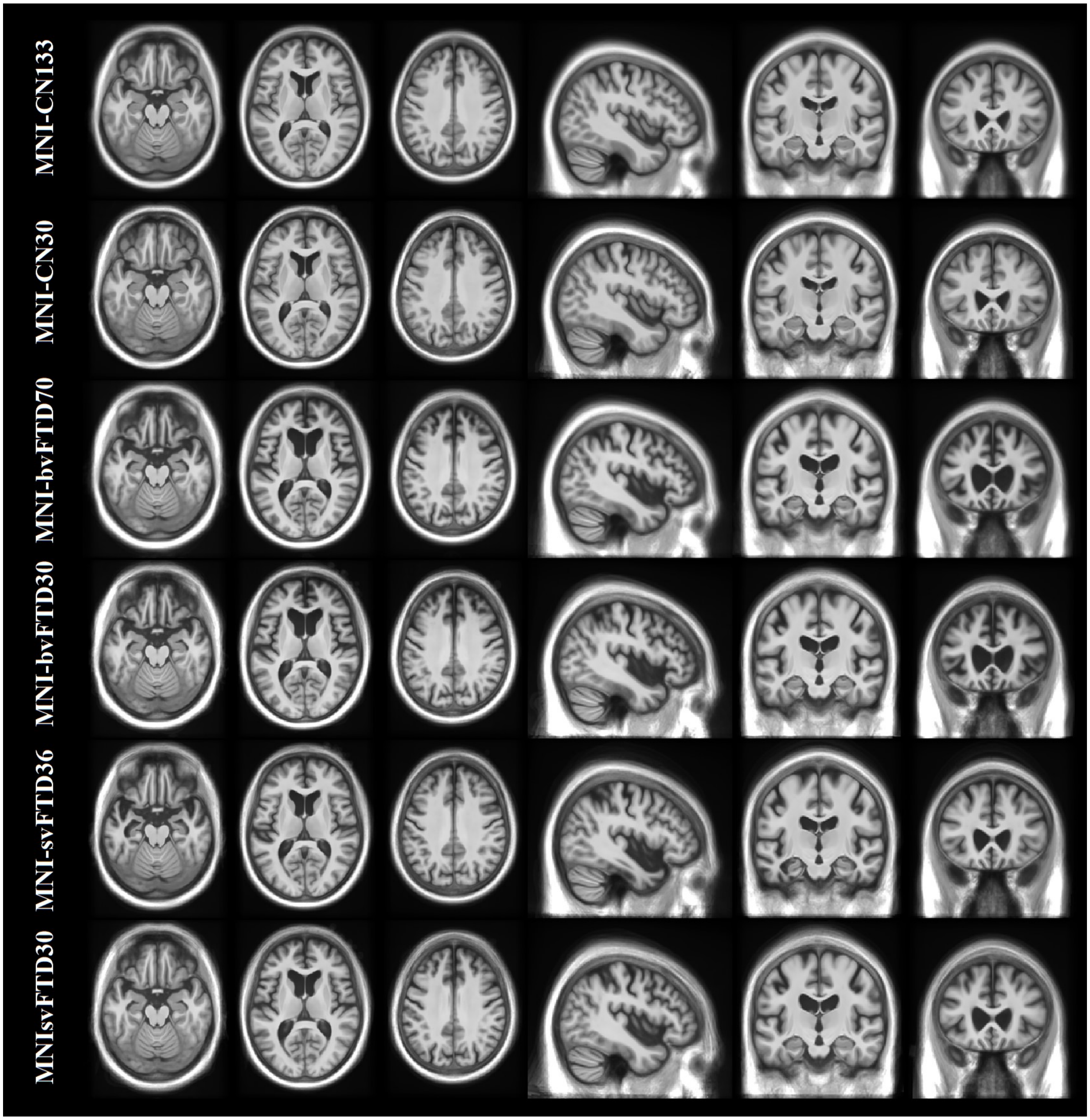

Figure S.12. MNI-CN133, MNI-CN30, MNI-bvFTD70, MNI-bvFTD30, MNI-svFTD36, and MNI-svFTD30 average templates. The figure shows predominant anterior frontal atrophy in the FTD templates compared to controls, more evident for the MNI-bvFTD70 and MNI-bvFTD30, whilst MNI-svFTD36 and MNI-svFTD30 templates show preponderant left anterior temporal atrophy.
